# Education note: cultivating assessment and feedback for learning in our training hospital

**DOI:** 10.1007/s13246-026-01721-7

**Published:** 2026-03-23

**Authors:** Rebecca Day, Louise Beckingsale

**Affiliations:** 1 Health New Zealand, Wellington Hospital, Wellington, New Zealand; 2 University of Otago, Christchurch, New Zealand

**Keywords:** Education, Training, Assessment, Feedback, Programmatic assessment, Radiotherapy

## Abstract

High-value feedback is essential for the successful development of medical physics trainees (registrars) as they complete their clinical training. In this education note, we present an overview of a small education intervention we implemented in our hospital to encourage feedback conversations, one of the principles of high-value feedback. Our intervention was based on the concepts of ‘assessment for learning’ and ‘programmatic assessment’. This intervention encourages registrars to take ownership of the feedback process associated with each learning outcome assessment. Registrars are encouraged to complete and document a self-assessment of their performance, before seeking feedback from their trainer. We modified an assessment template to support this process. This new approach appears to have resulted in higher quality feedback conversations between registrars and trainers. Additionally, trainers have benefited from the registrars completing the majority of the assessment template, thus reducing their workload. Whilst this approach has increased the workload for registrars, we believe this has benefits for their learning. We hope other centres can learn from our experience and may consider how they can adopt a similar process for their setting.

**Supplementary Information:**

The online version contains supplementary material available at 10.1007/s13246-026-01721-7.

## Introduction

*Assessment for learning* is an educational concept describing how, through the assessment process, learners build a picture of their own strengths and weaknesses, and thus optimize their own learning [1]. In recent decades this concept has become central to the design of educational programmes and stems from a change in thinking about the role of assessment in education. The more traditional approach that focussed on assessment of learning (for example, an examination that takes place at the end of instruction) has shifted to one where the assessment is inextricably embedded within the educational process [1]. This approach has proven to be successful within medical and veterinary schools where assessment is used intentionally for facilitating learning [2–4]. Assessment for learning places emphasis on feedback that guides learning and, for this feedback to be of high value, it is necessary that the ownership of the learning and feedback becomes the role of the learner rather than the role of the trainer [5, 6]. Self-assessment is an important part of this, helping to develop self-regulated learners who plan, evaluate, and monitor their own learning strategies [6].

In this education note, we share our experience of making small changes to the established assessment template to support radiation oncology medical physics trainees (known as ‘registrars’ in Australasia) to take ownership of the assessment and feedback process, and develop skills in self-assessment. In this education note, we use both ‘I’ and ‘we’ intentionally. ‘I’ is used when the first author (RD) is describing her personal insights, experiences, and reflections as a clinical supervisor in a training centre. ‘We’ is used when referring to the shared aspects of the project that were developed collaboratively between the first author (RD) and second author (LB) as project supervisor. These shared aspects included designing the proposed solution to support change, what we trialled, and what we learned about assessment for learning. This approach reflects the dual nature of the project as both an individual learning journey and a co-produced piece of scholarly work.

## Background

The Australasian College of Physical Sciences and Engineering in Medicine Training, Education and Assessment Program (ACPSEM TEAP) is a three year program in which postgraduate physicists train in a hospital-based centre to develop the skills and knowledge required to safely practice as certified medical physicists. In this paper, the focus is on training for radiation oncology medical physicists. The ACPSEM TEAP was revised in July 2022 to incorporate a programmatic model of assessment [7]. This model recognises that competency develops over time and gathers assessment information from multiple low stakes assessments by multiple assessors [8]. During the program, registrars are assessed by hospital-based trainers on learning outcomes across ten key curriculum areas. The ACPSEM provides assessment templates to document these assessments. The assessment templates commence with a section to describe the activity/task, the report topic, or the questions asked. The main body of the template consists of a table that is completed with either ‘falls short of expectations’, ‘meets expectations’ or ‘exceeds expectations’ for various criteria. For example, the oral assessment criteria are ‘knowledge of medical physics principles’, ‘communication’, and ‘application of relevant theory to clinical situations’. Additionally, there is space for a text comment for each criterion. Use of the templates is optional, and there is no requirement that they be completed by the trainer rather than the registrar. Within our centre, completion of the template has always been considered part of the trainer’s role, based on a more traditional bias towards assessment of learning and perhaps reinforced by the summative signals in the template wording.

Programmatic assessment draws on the principles of assessment for/as learning [1, 6, 9]. Each assessment provides the learner with an opportunity to develop an understanding of their strengths, weaknesses and areas for improvement. These low stakes assessments are decoupled from any decisions about a learner’s progression through the stages of the training program [9], which allows the focus to remain on high-value feedback rather than on decisions about progression.

Despite a growing body of evidence to suggest a programmatic assessment model supports assessment for learning [10, 3, 11], it comes with a number of challenges [12]. Two challenges that appeared relevant in my training centre were:

1. An increased assessment load and associated administrative workload related to an increased number of low stakes assessments, and,
2. A tendency for low stakes assessments to still be viewed as summative—emphasising pass/fail decisions rather than focussing on meaningful feedback, which can guide learning.

This project aimed to explore how these challenges affected the assessment of learning outcomes in my training centre, and to identify improvements that could strengthen an assessment for learning approach.

## Methods and solutions: discussions with the local training team

I met informally with three trainers and two current registrars in our training centre to discuss how they completed the learning outcome assessments. I also invited them to share their views on the purpose of the assessment templates, how they approached the assessment and feedback process, and how long the process took.

From these conversations, it was evident that the trainers, rather than the registrars, *lead* the assessment process. The assessment load was not perceived to be a major issue, but both registrars and trainers viewed the information on the assessment templates as primarily summative, i.e., the information being put on the assessment template focussed on whether the registrar had met expectations (often perceived as *passed)* or fallen short of expectations (often perceived as *failed)*, rather than the information being used to guide learning towards competency. Feedback was being provided by other means, but the assessment template itself was not being used to document this feedback. I additionally noted that, when feedback associated with the assessment was conducted, it was generally perceived as the trainer *giving* the registrar feedback rather than there being a two-way feedback conversation.

Overall, these discussions suggested that while the administrative workload was not perceived to be a significant concern it was clear the assessment templates could be used more effectively to enhance assessment and feedback for learning.

## Methods and solutions: what we trialled

Based on discussions with the registrars and trainers, and a review of relevant educational literature, we developed an education intervention to trial.

This intervention had three elements. Firstly, we aimed to place the registrar at the centre of the assessment and feedback process. “What the student does is actually more important in determining what is learned than what the teacher does” [13] is a well-known quote about learning and we therefore decided to position the registrar as the *owner* of that and a *seeker* rather than a *receiver* of feedback. Secondly, we wanted to incorporate self-assessment into the assessment and feedback process to develop self-regulated learning, critical reflection, and life-long learning [6]. Thirdly, we wanted to switch the focus from performance to learning thus promoting a growth mind-set [14–16].

During the trial period, the registrar initiated the assessment and feedback process by documenting their self-assessment on the assessment template. We asked that, ideally, the registrar and trainer complete the assessment template together immediately after the training task. If this was not possible, the assessment template could be completed by the registrar on their own and discussed with the trainer at a later date.

For each section of the assessment template, the registrar was asked to complete a self-assessment outlining what they thought they did well, how well they understood the task/topic, and what they could improve. They also identified areas where they sought specific feedback. The trainer then responded to each of these points, including offering strategies for improvement and action points.

**Fig. 1:**
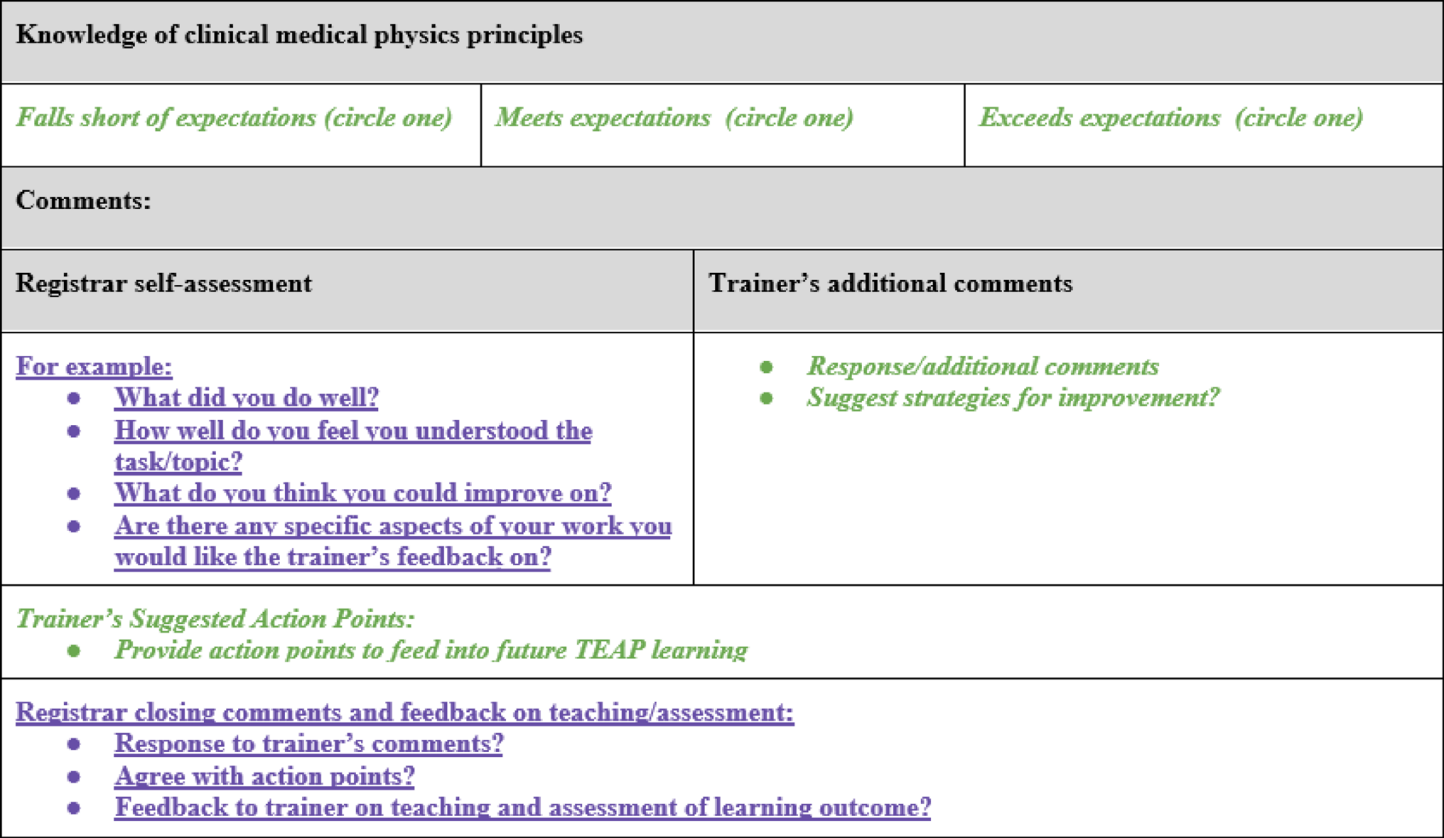
Outline assessment template that was provided to the team to show the preferred format. The sections the registrar fills in are shown in purple underlined text. The sections the trainer fills are shown in green italic text

The modified assessment template is shown in Fig. 1. An exemplar assessment template (online resource 1) was also provided to the team illustrating how the template might be completed for a hypothetical assessment.

We hoped that this new approach would not increase the assessment workload of the trainer and might even reduce it because the template could be completed by or with the registrar. I introduced the concept to the team in a tutorial, which included a mock oral assessment so the team could practice applying the new approach.

## What have we learned? What has been successful?

### The positives we have observed

The new approach for completing assessment templates has been in use in our team for over 18 months. I have noticed that changing the way the assessment templates are completed has made them a more effective tool to facilitate a feedback conversation between trainers and registrars. Having the registrar initiate the completion of the assessment template by first reflecting on how well they did and how they could have improved, has positioned them as the seeker of feedback, which enhances the effectiveness of the feedback [17]. This process encourages self-regulated learning by promoting self-assessment and self-reflection - skills essential for life-long learning [6]. Further, once the registrar has completed their training, these skills can be directly applied to their continuing professional development (CPD), which is mandatory for ACPSEM certification.

The new process also appears to have shifted the assessment away from being a summative process to one that guides future learning. This aligns with a constructionist theory of learning, which places the importance on what the student does rather than what the teacher does [13].

One practical benefit of this change is that registrars no longer have to chase trainers to complete the assessment template. Trainers now receive a template that is mostly completed, making the task quicker and more appealing. When the trainer agrees with the registrar’s self-assessment, minimal additional input is required.

Finally, this approach has improved the documentation of feedback. Prior to this change, feedback was given verbally, by email, or comments on reports, but not consistently recorded on the assessment templates. Documenting feedback within the templates now provides the ACPSEM with a more complete view of each registrar’s progress.

Our positive findings are consistent with many other studies. Heeneman et al. [2] reported that most students in a medical school context perceived reflective writing in a portfolio as valuable for learning from the feedback and for the self-regulation of learning. Liu et al. [18] showed that, whilst it takes time to adapt to new assessment approaches, students of an online master’s programme for health professionals perceived programmatic assessment, including self-assessment in e-portfolios, as enhancing their learning experience.

### The challenges we have observed

Whilst we believe the feedback conversations facilitated by the modified assessment template have improved, we acknowledge the workload for the registrar has increased. However, we believe this increased workload has been worthwhile for the enhanced learning and feedback that has resulted.

Some team members noted that the self-assessment component can feel ‘awkward’ and ‘difficult’ for the registrar. However, we view this as a valuable part of the learning process because developing the ability to self-assess is an important, albeit challenging, metacognitive skill [6]. This concern around the awkwardness of self-assessment came from some of the trainers rather than the registrars themselves, and we have not actually encountered any reluctance from the registrars to engage in self-assessment. ACPSEM have implemented the use of reflective documents for other parts of TEAP training, so the registrars have become practiced at reflection which is an important part of self-assessment.

A few trainers found it difficult to provide action points for future learning. They tended to view each learning outcome assessment as discrete rather than supporting progressive development towards the desired standard or competency. To help clarify the purpose of feedback, we modified the wording on assessment template from ‘Provide action points’ to ‘Provide action points to feed into future TEAP learning’.

This change in practice is not compulsory and I have noticed that some trainers continue to complete the templates without encouraging the registrar to complete their self-assessment first. I will continue to encourage trainers and registrars to employ this new approach by role modelling this approach in my own practice and including it in orientation of new registrars and team members. I have primarily focussed on persuading the registrars of the benefits of this approach, so that they choose to take the lead in the assessment process. I acknowledge that our trainers are prioritising a highly demanding clinical workload, and may not have received any professional development in education or assessment. I believe some flexibility is needed in allowing the trainers to complete the templates whichever way they see fit, whilst working behind the scenes with the registrars to encourage them to lead the process and take the strain off the trainers.

### What are the next steps?

We acknowledge that completing assessment templates involves a high administrative workload and that this new approach has shifted most of this workload to the registrar. Moving forward, we plan to review the modified assessment template through an audit of how it has been employed since being introduced. This audit may identify sections of the template that are not well used, and may allow us to simplify the format. This may help to streamline the process whilst still maintaining the positive learning outcomes.

I also acknowledge that this is the first step in changing the assessment and feedback culture in my training centre. I am keen to grow the team’s understanding of assessment for learning by offering a series of education-focussed seminars that promote discussion about the new approach and continue to strengthen our feedback and learning culture.

We recognise that this approach relies on a strong, trusting, and open relationship between registrars and trainers [17]. Our small, close-knit team develops such relationships naturally over the three years of a registrar’s training, providing a solid foundation for this approach to succeed. We acknowledge that the approach might work less effectively at the start of the training, when these trusting relationships have not had much time to develop. One option for consideration, would be to encourage use of this approach only after a certain time-period or milestone, for example, after the first clinical and scientific report submission that is due 9 months after the registrar commences clinical training.

While this approach might not be as easily adopted in other centres, it offers transferable lessons. Education interventions often need to be adapted to local context and we encourage other TEAP teams to trial and modify for their setting, and share their experiences. Positioning registrars as the owners of the assessment and feedback process has clear educational benefits. Even if assessment template does not follow our prescribed format, having the registrar lead the assessment and feedback process could be easily adopted by other centres.

## Conclusion

We introduced a new way for completing assessment templates in our training centre to support an assessment for learning approach. This approach has placed the registrar as the owner of the assessment and feedback process, enhancing the value of feedback, and supporting them to develop skills in self-assessment. Although this approach has increased the registrars’ workload, it empowers them to lead the feedback process and ensures the templates are completed in a timely manner. We believe this approach has started to change the feedback culture in our team by reframing the completion of the assessment template as a collaborative feedback conversation between registrar and trainer.

## Supplementary Information

Below is the link to the electronic supplementary material.

Supplementary Material 1

## Data Availability

As a reflective piece, a data availability statement is not applicable to this work. However, data related to the study are available from the corresponding author on reasonable request.
