# Supplementary material for: Education note: cultivating assessment and feedback for learning in our training hospital
Source: Phys Eng Sci Med. 2026 Mar 23;49(2):1105–10. doi: 10.1007/s13246-026-01721-7 (PMC13375686; doi:10.1007/s13246-026-01721-7)
Supplement: Supplementary file 1 — Supplementary Material 1 [file 13246_2026_1721_MOESM1_ESM.docx]

# Assessor Guide: Oral Assessment

Use this template document to complete assessment for a Learning Outcome or Element that requires an oral assessment. Circle or mark the appropriate level (based on the assessment rubric) for each criterion for each question or topic discussed. A separate table can be used for individual questions/topics as needed.

## Learning outcome / element

**E 3.1.5a - How calibration factors are transferred from the PSDL to the department**

## Question asked or topic discussed

**Describe how the MV x-ray dose measured at a linac with a field instrument is traceable to the international standard for absorbed dose?**

**Follow up questions (if not covered in registrar response):**

- **Why is it important that we maintain our traceability of calibration?**
- **What standards’ lab do we use?**
- **What beam energy or energies are used at our standards’ lab when they calibrate our reference chamber?**
- **What sort of dosimeter is the primary standard device at our standards’ lab?**
- **What is the unit of measurement used for the calibration of our department reference chamber?**
- **How often is the department reference chamber for MV photons sent to the standards’ lab?**
- **Describe the steps taken to cross calibrate a department field instrument against the department reference chamber for MV Photons?**

**If the local standards’ lab announced that they found a systematic error in all their MV x-ray calibrations from the last 10 years of 0.5%, what consequences would this have had for patients treated at our centre during this time, and also more generally for radiotherapy services in the region?**

## Oral Assessment Rubric

| **Knowledge of clinical medical physics principles** | | |
| --- | --- | --- |
| **Falls short of expectations** | **Meets expectations** | **Exceeds expectations** |
| **Comments:**   \| **Registrar self-assessment:** \| ***Trainer additional comments:*** \| \| --- \| --- \| \| - **Overall I think I correctly covered most of the points you would have expected in an answer to this question, although my trainer did need to prompt me to ask about the energies used for calibration at the standards’ lab.** - **I think I understand the importance of traceability of calibration, and the fundamentals of how this is carried out in practice.** - **I did get a bit confused when discussing the energies used for the calibration at the standards’ lab. I need to do some more reading around that and get it completely straight in my head because I got mixed up between the approach when the reference chamber is calibrated in Co-60 and when a range of linac beams are used for the calibration, especially about how department cross calibrations are conducted with the two approaches. I’d like some help to clarify my understanding of this.** \| - ***Agreed. You only needed prompting about some very specific details.*** - ***Agreed.*** - ***Agreed. I can provide some reading material from the ARPANSA reference dosimetry course which may help clarify your understanding. I can also point you towards some past exam questions from previous written exams which you should try and answer and this should help you understand.*** \|   ***Action points:***   - ***Reading of APRANSA reference dosimetry course notes that I will provide*** - ***Past exam questions to be provided.***   ***TRAINER’s Grade: Meets expectations.***  **Registrar closing comments and feedback on the teaching/assessment:**   - **Thanks for the reading and past exam questions: that will definitely help.** - **The reading material you gave for LO was generally good, although there was rather too much of it! You set some questions for me to do written answers for prior to our tutorial, and then we discussed my answers in the tutorial. The questions were a really good way to make me think about the reading material. The formal oral assessment was good, but it was three weeks after our tutorial. It would have been better if the oral assessment followed more quickly after the tutorial.** | | |
| **Communication** | | |
| **Falls short of expectations** | **Meets expectations** | **Exceeds expectations** |
| **Comments:**   \| **Registrar self-assessment:** \| ***Trainer additional comments:*** \| \| --- \| --- \| \| - **Overall I think I communicated my answers clearly and logically.** - **I did get in a bit of a muddle with my response when I became confused about the beam energies used for calibration. I’d like to know how I could have handled that better.** \| - ***Your oral communication was mostly good: You were confident in your answers and articulate. However, you didn’t always answer in the most logical order. Sometimes you answered in a very roundabout fashion: you covered all the points but not always very succinctly!*** \|   ***Action points:***   - ***I think you need to do more practice answering these sorts of questions. Could you come up with a ‘template’ to have in your head for giving an answer e.g. (1) talk about what documents/guidelines you’d consult to answer the question (2) give a broad overview of the concept/process (3) talk through each step in the concept/process giving the relevant practical details you think the examiner is expecting.*** - ***Please pair up with a fellow registrar (from another centre?) and use the questions from the oral assessment to practice giving answers to each other.***   ***TRAINER’s Grade: Meets expectations.***  **Registrar closing comments and feedback on the teaching/assessment:**   - **A template answer format is a good idea: I’ll try that out when I practice with another registrar.** | | |
| **Application of relevant theory to clinical situations (if applicable)** | | |
| **Falls short of expectations** | **Meets expectations** | **Exceeds expectations** |
| **Comments:**   \| **Registrar self-assessment:** \| ***Trainer additional comments:*** \| \| --- \| --- \| \| - **I wasn’t expecting the question about the clinical consequences of a systematic mis-calibration by the standards’ lab, so I floundered a bit.** \| - ***You made some good points when answering this question, but you mostly focussed on what the consequences might be for individual patients. It would have been nice if you’d talked a little bit about how a systematic deviation might affect the results we report in NZ relative to the rest of the world, and might have an influence on clinical trial results. Nether-the-less, the answer was good considering your training stage.*** \|   ***Action points:***   - ***None. Your ability to discuss the application of theory to clinical situations will grow with experience.***   ***TRAINER’s Grade: Meets expectations.***  **Registrar closing comments and feedback on the teaching/assessment:**   - **None** | | |

## Question asked or topic discussed

## Oral assessment rubric

| **CRITERION** | **FALLS SHORT OF EXPECTATIONS** | **MEETS EXPECTATIONS** | **EXCEEDS EXPECTATIONS** |
| --- | --- | --- | --- |
| **Knowledge of clinical medical physics principles** | - Demonstrates an understanding of most theory, but some weaknesses still present - Demonstrates an understanding of how the theory is used to guide clinical practice, but still needs prompts from Supervisor | - Demonstrates a strong understanding of relevant theory - Independently demonstrates an understanding of how the theory is used to guide clinical practice | - Demonstrates an extensive knowledge of theoretical concepts - Uses innovative thinking to suggest improvements to clinical practice |
| **Communication** | - Unable to clearly demonstrate sound communication skills - Questions required by Supervisor to clarify the meaning of some answers | - Demonstrates sound scientific communication with only minor deficiencies. Mostly confident, articulate, and logical oral scientific communication | - Demonstrates proficiency in scientific communication with no deficiencies. Very confident, articulate, and logical oral scientific communication |
| **Application of relevant theory to clinical situations** | - Demonstrates only a basic understanding of why tasks are performed | - Demonstrates an understanding of the rationale and purpose behind all work performed - Demonstrates the ability to discuss non-routine processes with the supervisor | - Demonstrates the ability to critique routine procedures - Demonstrates the ability to describe non-routine processes independently |
